# Exploiting Protein N-Terminus for Site-Specific Bioconjugation

**DOI:** 10.3390/molecules26123521

**Published:** 2021-06-09

**Authors:** Lucia De Rosa, Rossella Di Stasi, Alessandra Romanelli, Luca Domenico D’Andrea

**Affiliations:** 1 Istituto di Biostrutture e Bioimmagini, CNR, Via Mezzocannone 16, 80134 Napoli, Italy; lucia.derosa@cnr.it (L.D.R.); rossella.distasi@cnr.it (R.D.S.); 2 Dipartimento di Scienze Farmaceutiche, Università Degli Studi di Milano, Via Venezian 21, 20133 Milano, Italy; alessandra.romanelli@unimi.it; 3 Istituto di Scienze e Tecnologie Chimiche “Giulio Natta”, CNR Via M. Bianco 9, 20131 Milano, Italy

**Keywords:** protein labeling, chemo-selective reaction, molecular probe, chemical ligation, click-chemistry, aldehyde protein, azide protein

## Abstract

Although a plethora of chemistries have been developed to selectively decorate protein molecules, novel strategies continue to be reported with the final aim of improving selectivity and mildness of the reaction conditions, preserve protein integrity, and fulfill all the increasing requirements of the modern applications of protein conjugates. The targeting of the protein N-terminal alpha-amine group appears a convenient solution to the issue, emerging as a useful and unique reactive site universally present in each protein molecule. Herein, we provide an updated overview of the methodologies developed until today to afford the selective modification of proteins through the targeting of the N-terminal alpha-amine. Chemical and enzymatic strategies enabling the selective labeling of the protein N-terminal alpha-amine group are described.

## 1. Introduction

Site-specific protein bioconjugation refers to the controlled modification of the protein chemical structure through the introduction of functional handles at well-defined positions along the protein sequence, endowing such biomolecules with novel and useful properties. A wide set of site-specific protein bioconjugation chemistries have been developed up to today, allowing for the effective preparation of modified proteins with valuable applications that pave the way to relevant scientific innovations in many disciplines of the life sciences such as in chemical biology, biophysics, biotechnology, material sciences, and biomedicine [1,2,3,4,5]. In these research fields, the use of site-specific protein decoration strategies instead of stochastic labeling methodologies is strictly required in order to ensure the highest level of uniformity over the properties of the protein conjugates, especially in the case of protein pharmaceuticals. In this latter context, several protein molecules site-specifically decorated with imaging probes, drugs, or carriers have been developed as novel protein-based diagnostic and therapeutic tools and many of them are successfully proceeding in clinical trials or are still to reach the pharmaceutical market [6,7,8]. Conventional strategies used to modify a protein molecule in a site-specific manner target amino acid side-chains [2,9], exploiting the peculiar reactivity of unique functional groups such as the thiol of cysteine [10], the epsilon-primary amine of lysine [11,12], the thioether of the methionine [13,14,15], the indole [16,17], imidazole [18], and phenol ring [19] respectively of tryptophan, histidine, and tyrosine. However, such methodology strictly requires the presence of a single reactive amino acid along the protein sequence to avoid multiple and heterogeneous patterns of decoration.

A more sophisticated and laborious methodology affording site-specific protein labeling relies on the use of the expanded genetic code to introduce by recombinant expression an unnatural amino acid into a protein target, which can react with orthogonal probes through chemo-selective reactions [20,21]. Although representing a noteworthy technological advance, such an approach is technically demanding and low-yielding and, therefore, does not provide a resolutive solution to the issue. Chemical ligation approaches, in particular native chemical ligation (NCL) [22] and expressed protein ligation (EPL) [23], consisting of the assembly of a protein from synthetic or recombinant peptide segments, have revolutionized the protein bioconjugation concept and scope, allowing us to virtually introduce any kind of modification with surgical site-specificity [24,25]. However, chemical ligation reactions are usually performed in denaturing conditions and thus the labeled protein requires a refolding step that is not always successful and high-yielding. The search for novel bioconjugation chemistries point to overcoming such limitations, aiming at developing methodologies that ensure (1) a high level of selectivity, (2) could be performed in mild conditions compatible with the preservation of the native protein structure and function; (3) could be easily scaled-up; and (4) could be generally applicable to any protein target. Protein labeling through the selective targeting of the protein N-terminal alpha-amino group has recently emerged as a convenient solution to this issue, allowing to potentially satisfy all the above-mentioned requirements [26]. The N-terminal alpha-amino group is universally present in each protein molecule and is a unique functionality within a polypeptide chain endowed with a peculiar reactivity that allows it to be chemically distinguished from the other nucleophiles harbored by a protein molecule (i.e., the epsilon-amine group of lysines and the thiol group of cysteines). In fact, the N-terminal amine group is the only alpha-amine present within a protein molecule. It possesses an adjacent amide bond that influences its reactivity by lowering its pKa value (6.0–8.0) with respect to that of the epsilon-amine group of lysine (~10.5) and to the thiol group of the cysteine (~8.3) [27]. Consequently, the alpha-amine group is the most reactive nucleophile within a protein molecule at pH around neutrality and, therefore, can be selectively targeted with electrophilic probes at physiological pH. However, it should be noted that the pKa value of a functional group within a native protein molecule could in some cases vary from the expected one due to the influence of the chemical environment. Protein N-terminal alpha-amine is usually exposed on the protein surface, thus resulting in being easily accessible to chemical modification [28]. Accordingly, protein modification at this position usually has minimal or no impact on protein structure and function. For all these reasons, N-terminal protein labeling has gained great interest, leading in recent years to the development of a portfolio of chemical and enzymatic strategies for selectively tagging such protein sites. Herein, we provide a comprehensive overview of the bioconjugation chemical strategies developed to selectively target protein N-terminus using direct or indirect labeling procedures (Table 1). Direct approaches provide N-terminal protein modification in one step, taking advantage of molecular probes able to selectively react with such protein sites, often through conjugation reaction mechanisms that involve the side chain of the first amino acid of the protein sequence. Indirect approaches are, instead, two-steps procedures in which the N-terminus is first converted into a reactive handle and then selectively targeted with an orthogonal probe through a second reaction step. Although one-step approaches for direct protein labeling may appear more convenient and rapid, indirect strategies are more versatile with respect to the kind of probe to be introduced as they may take advantage of the wide collection of commercially available molecular probes without requiring severe synthetic efforts. Enzymatic approaches affording N-terminal protein labeling are also summarized (Table 2).

## 2. Chemical Strategies Targeting Protein N-Terminus

### 2.1. Direct Labeling of Protein N-Terminus

**Figure 1:**
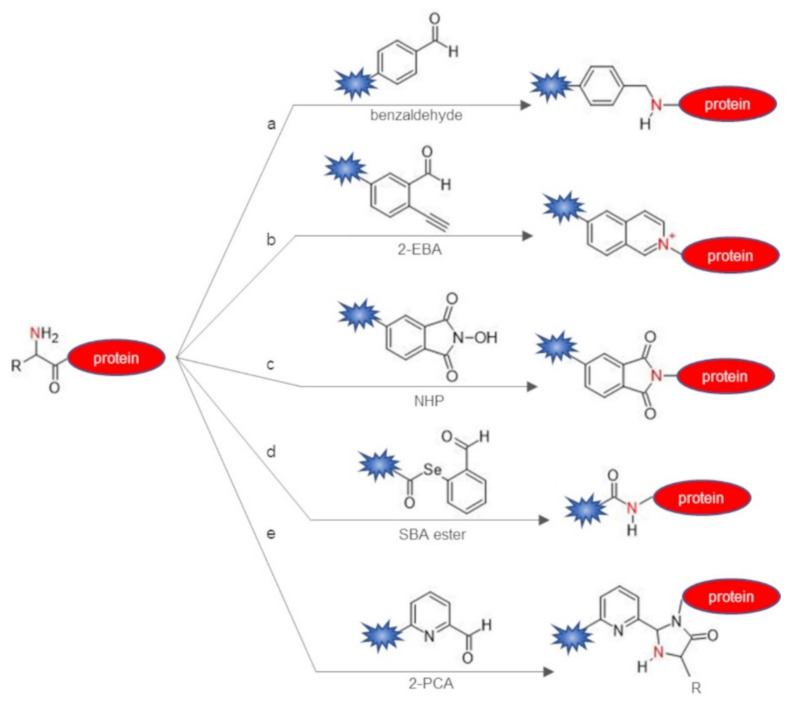
Selective protein labeling through the direct targeting of the N-terminal alpha-amine group. (**a**) Reductive amination with a benzaldehyde probe; (**b**) imine formation and intramolecular 6-endo-dig cyclization with a 2-ethynylbenzaldehyde (2-EBA) probe; (**c**) phthalimidation with a N-hydroxy-phthalimide (NHP) probe; (**d**) aldehyde capture ligation with a selenobenzaldehyde (SBA) ester probe; (**e**) imine condensation with a 2-pyridinecarbaldehyde (2-PCA) probe.

The simplest approach used to directly target protein N-terminus refers to the use of amine-reactive compounds such as activated ester, for example, N-hydroxysuccinimide (NHS) esters or perfluorophenyl esters, exploiting the different pKa value of the alpha-amine to discriminate between the N-terminal amine group and the other nucleophiles by performing the labeling reaction at low-to-neutral pH [51]. NHS ester probes react in mild conditions with the N-terminal amine groups through an acylation reaction, leading to the formation of an amide conjugate, as shown using unprotected peptides [52]. Aldehydes may also be used to selectively target N-terminal alpha-amine in a pH-controlled manner using different chemistries. Benzaldehyde derivatives have been used to selectively alkylate the protein N-terminus by reductive amination in the presence of sodium cyanoborohydride (NaBH_3_CN), leading to the formation of a secondary amine as the conjugate product [29] (Table 1, Figure 1a). Conveniently, protein modification by reductive amination allows the positive charge on the N-terminus to be preserved, which could be crucial to retain, in some cases, protein bioactivity. Similarly, 2-ethynylbenzaldehyde (2-EBA) derivatives were reported to selectively react with the protein N-terminal alpha-amine group in phosphate-buffered saline solution at pH 6.5–7.4 through the formation of an isoquinolinium conjugate in which the nitrogen atom is positively charged (Figure 1b) [30]. A phthalimidation protocol using N-hydroxy-phthalimide reagents and targeting protein N-terminus has also been reported, showing selectivity against alpha-amine in the presence of lysine residues at pH 7.0 (Figure 1c) [31]. The reaction afforded a phthalimidoamine product that can be conveniently converted back to the amine in the presence of hydrazine. Selenobenzaldehyde esters are also able to selectively react with the N-terminal alpha amine in the presence of multiple Lys residues in a pH-controlled manner through aldehyde capture ligation (ACL), which consists of an acylation reaction that yields an amide bonded conjugate [32] (Figure 1d).

**Figure 2:**
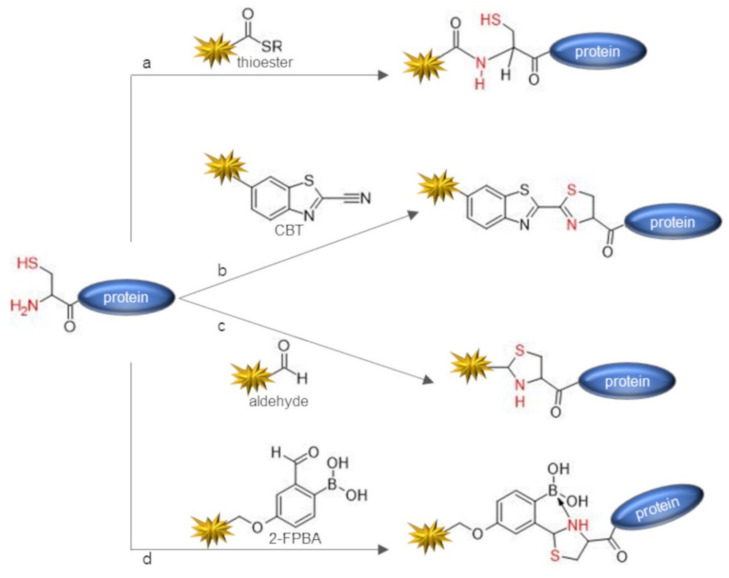
Selective targeting of N-terminal cysteinyl proteins. (**a**) Native chemical ligation with a thioester probe; (**b**) condensation with a 2-cyanobenzothiazole (CBT) probe; (**c**) thiazolidine ligation with an aldehyde probe; (**d**) condensation with a 2-formyl phenylboronic acid (2-FPBA) probe.

However, labeling approaches based on the control of the pH to target protein N-terminus do not ensure complete selectivity and off-target labeling may frequently occur, leading to heterogeneous protein mixtures. Most sophisticated methodologies developed to directly target protein N-terminus exploit the selective reactivity of small organic molecules against the N-terminal alpha-amine by involving the participation of the side-chain of the first amino acid in the conjugation reaction mechanism. Several approaches have been developed to target the 1,2-aminothiol function exposed by N-terminal cysteinyl proteins (Figure 2).

**Figure 3:**
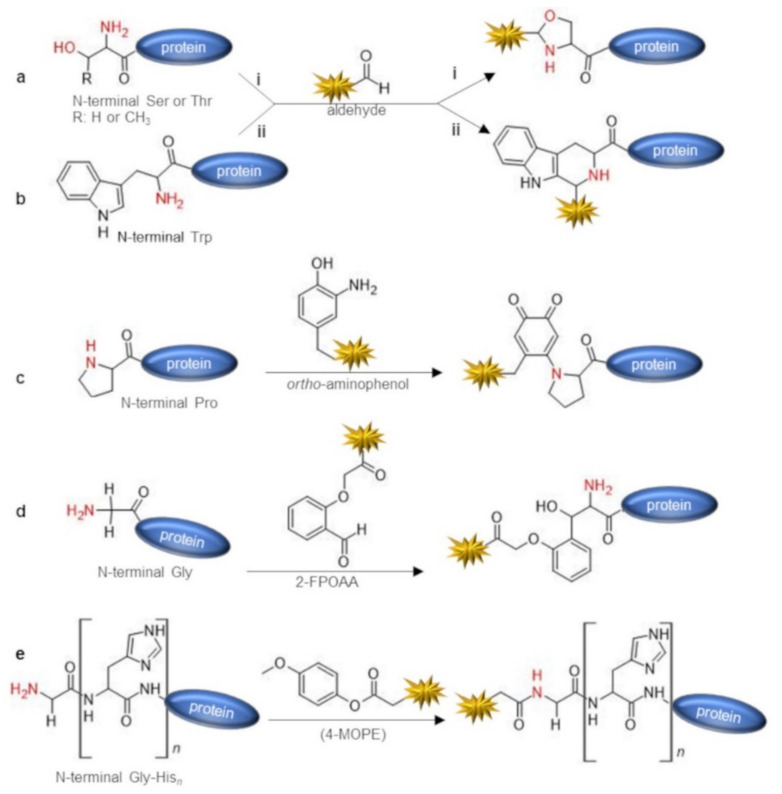
Site-specific protein labeling through the targeting of N-terminal Ser (or Thr) (**a**), Trp (**b**), Pro (**c**), Gly (**d**), or Gly-His_n_ (**e**).

Cysteines are rarely present at the N-terminal position in natural proteins and therefore should be introduced by protein engineering [53]. However, the additional Cys residue may hamper the correct pairing of native Cys residues into disulfide bridges, and hence may lead to protein misfolding. Besides, during recombinant expression and protein purification, the N-terminal cysteine may undergo the cyclization reaction by condensation with aldehyde catabolites present within the cell, leading to the formation of a thiazolidine derivative that masks the terminal 1,2-aminothiol function [54]. Although in this case the N-terminal cysteine could be restored by treatment with O-methylhydroxylamine [54,55], the most convenient strategy to prepare N-terminal cysteinyl proteins refers to the exposition of the N-terminal cysteine after protein expression and purification through proteolytic cleavage with Factor Xa, tobacco etch virus (TEV), and thrombin proteases [56,57,58] or using self-cleavable fusion partners such as intein [59]. The most powerful and widespread chemical approach exploited to target N-terminal cysteinyl proteins refers to the reaction with a thioester probe through NCL [33,54,60,61,62,63,64,65] (Figure 2a). The NCL reaction proceeds through a reversible trans-thioesterification mediated by the thiol group of the cysteine, followed by a spontaneous intramolecular S→N acyl shift that translocates the probe on the alpha-amine group, affording an amide bonded product [22]. Notably, the thiol group of the N-terminal cysteine mediates NCL reaction but is restored in the free form in the final conjugated product. Therefore, such a thiol group could be exploited for pursuing second site-specific labeling using a thiol reactive probe, for instance, through the thiol–maleimide chemistry [64,66]. Conveniently, thioester probes can be obtained from commercially available NHS-ester derivatives by treatment with thiols [65,67]. N-terminal cysteine can also be directly tagged using 2-cyanobenzothiazole (CBT) through a water-compatible condensation reaction [34] that mimics the last reaction step in the synthesis of D-luciferin in firefly [68] (Figure 2b). The reaction is fast, requires slight excess of the CBT-probe, leads to a stable conjugate, and efficiently proceeds in aqueous solution at physiological conditions (PBS pH 7.4), also being compatible with in cells and in vivo imaging applications [34,69,70]. The ability of N-terminal cysteine to react with aldehydes through a condensation reaction forming a thiazolidine derivative has also been explored as a N-terminal labeling strategy [71,72] (Figure 2c). N-terminal serine and threonine may similarly react with aldehyde probes, providing an oxazolidine conjugate [73] (Figure 3a).

Although thiazolidine ligation has been successfully applied to the synthesis of protein conjugates [35,74], thiazolidine ligation suffers of severe limitations as the reaction requires a slightly acidic pH (pH 4–5), is not always well tolerated by the protein target, and shows a slow kinetic requiring several days of incubation to proceed, even in the presence of large excess of the aldehyde reactant. Besides, the thiazolidine product is not stable and may hydrolyze [26]. The use of ortho-boronic acid substituted benzaldehydes such as reagents such as 2-formyl phenylboronic acid (2-FPBA) allows us to overcome the drawbacks of thiazolidine ligation. 2-FPBA promotes the fast formation of a thiazolidino-boronate (TzB) conjugate by reacting with the N-terminal cysteine at neutral pH and in equimolar ratio between the protein and the probe derivative [36,75] (Figure 2d). Notably, the TzB product was found to exhibit superior stability at physiological pH with respect to thiazolidine conjugates due to boron coordination by the thiazolidine ring. Conveniently, TzB may dissociate in the presence of benzyl hydroxylamine [75] and at acidic pH [36]. This latter feature makes the TzB chemistry interesting for the development of pH responsive drug-conjugates such as antibody–drug conjugates designed to selectively release the cytotoxic payload in the endosomes. Aldehyde probes may also be exploited to target an N-terminal tryptophan-containing protein through the Pictet–Spengler reaction that generates a carbon–carbon bonded conjugate [37,76] (Figure 3b). Such a reaction was performed to ligate peptide fragments in acid conditions and is not compatible with the preservation of the native protein structure. However, the Pictet–Spengler reaction was also performed in milder conditions, conjugating an indole probe on the N-terminal aldehyde protein [77,78]. Francis et al. reported a versatile and straightforward method that used 2-pyridinecarbaldehyde (2-PCA) based probes to selectively label protein N-terminal alpha-amine without requiring the assistance of the first amino acid side-chain [38] (Figure 1e). 2-PCA reacts with the protein N-terminal alpha-amine through an imine condensation reaction, after which the amide nitrogen of the neighboring amino acid cyclizes on the imine to form an imidazolidinone conjugate. As they lack the nearby amide group, the amine group of lysine side chains are not able to form stable products, thus ensuring high selectivity of the chemistry against the N-terminus. Apart from the N-terminal prolyl protein, which cannot react with 2-PCA, the method is general and proceeds smoothly in a variety of common biological buffers at pH 7.5. The imidazolidinone conjugate showed high stability over a pH range from 3 to 11 but was prone to hydrolysis at 37 °C. The repertoire of bioconjugation chemistries for the direct labeling of protein N-terminus also includes an oxidative coupling reaction performed at pH 7.5 with ortho-aminophenols catalyzed by potassium ferricyanide (K_3_Fe (CN)_6_), affording a stable bioconjugate [39] (Figure 3c). The reaction requires a slight excess of the ortho-aminophenol (2–5 eq). Although the majority of N-terminal amino acids showed good-to-high levels of conversion (60−90%), the reaction worked more efficiently on N-terminal prolyl peptides and proteins, reaching in this case nearly complete modification (90−100%) and reacting in a shorter time (less than 30 min). Reaction of an ortho-aminophenol with an N-terminal prolyl protein gives an ortho-quinone product that resulted stable to reductant, nucleophiles, and acid or basic pH. Disadvantageously, cysteine side-chains also react with o-aminophenols, limiting the applicability of the strategy to protein without cysteines or to their temporary protection with 5, 5′-dithiobis (2-nitrobenzoic acid) (DTNB; Ellman’s reagent). Very recently, an effective chemical platform for the targeting of N-terminal glycinyl proteins has also been reported [40] (Figure 3d). The great advantage of this methodology is that natural proteins often possess a N-terminal glycine and such residue can be easily exposed at the protein N-terminus during recombinant expression, resulting in a versatile and general labeling methodology [79,80]. In fact, the N-terminal formyl-Met (fMet) residue is promptly removed during recombinant expression of glycinyl-proteins by the intracellular Met-aminopeptidase, which efficiently acts on the nascent polypeptide chain to expose the N-terminal Gly residue. Besides, the protease cleavage sites introduced at the N-terminus of a recombinant protein, which allow for the removal of the affinity tag by site-directed proteolysis after purification such as TEV or Factor Xa cleavage sites, are usually designed to leave a Gly residue as the +1 amino acid of the protein target. The N-terminal glycine directed bioconjugation chemistry exploits ortho-substituted benzaldehydes such as 2-(2-formylphenoxy) acetic acid (2-FPOAA) with an appropriately designed carbonyl substituent as a hydrogen bond acceptor that results in the exclusive labeling of the N-terminal glycine residue, affording a stable amino-alcohol as a bioconjugation product through the intermediate formation of an imine. The reaction proceeds under mild conditions, being performed in a carbonate buffer at pH 7.8 at room temperature and was demonstrated to exclusively target the N-terminal glycine even on protein targets exposing lysine or cysteine residues. Using a symmetric bis-aldehyde derivative of the reagent, various chemical tags can be selectively introduced on the protein N-terminus exploiting the second aldehyde function for protein functionalization. Notably, such additional aldehyde function was also exploited for protein purification through hydrazone ligation using a hydrazide derivatized resin. The immobilized protein could be effectively released from the solid support by treatment with O-hydroxylamine derivatives through trans-oximization. Inspired by an undesired chemical modification occurring at the N-terminus of His-tagged recombinant protein [81], D-gluconic acid δ-lactone (GDL) was demonstrated to effectively acylate the alpha-amine group of protein targets harboring the on Gly-His_n_ N-terminal amino acid stretch [41]. A higher yield of N-terminal acylation was obtained for tag sequence containing at least three His residues. The reaction selectively proceeded in HEPES buffer at pH 7.5 with high yield but appeared to be reversible. Other esters resulted in being able to selectivity and irreversibly acylate the alpha-amine of the on Gly-His_n_ tag, in particular, the 4-methoxyphenyl ester was selected as the optimal reagent and conveniently modified with functional handles such as a biotin or an azide group (Figure 3e). The reason for the selective acylation of the N-terminal alpha-amine on Gly-His_n_-tagged protein resides in the base catalysis of the reaction mechanism in which the His side-chain assists the deprotonation during the direct acylation of the Gly alpha-amine. Conveniently, Gly-His_6_ tag, harboring six His residues, shows a dual application, as the His_6_-tag affords recombinant protein purification by affinity chromatography on Ni^2+^-nitriloacetic resins and can be exploited for N-terminal protein functionalization with GDL or 4-methoxyphenyl ester probes.

### 2.2. Indirect Labeling of Protein N-Terminus

**Figure 4:**
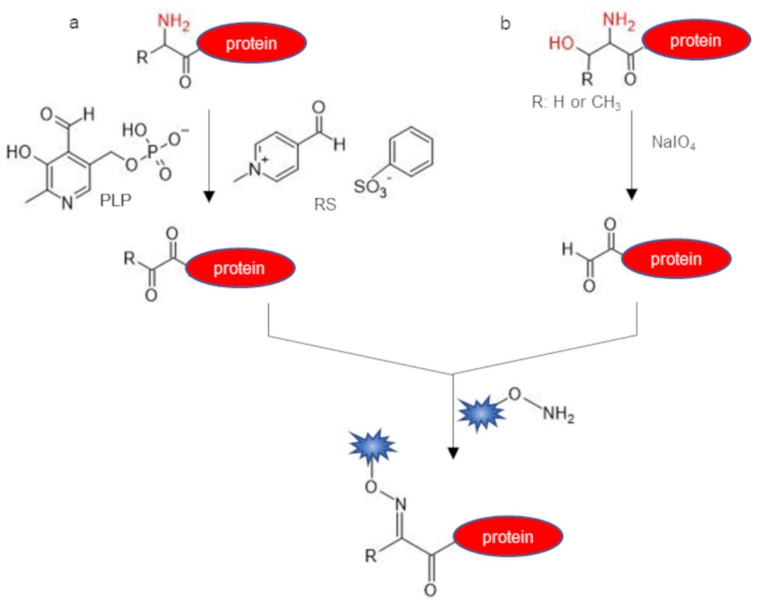
Two-step N-terminal protein labeling. (**a**) Transamination reaction with pyridoxal-5′-phosphate (PLP) or with N-methylpyridinium-4-carboxaldehyde benzenesulfonate salt (Rapoport’s salt, RS), leading to a N-terminal ketone protein; (**b**) oxidation with sodium periodate (NaIO_4_) of 1,2 amino alcohol (N-terminal Ser or Thr), affording a N-terminal aldehyde protein. The ketone/aldehyde function installed at the protein N-terminus can be further reacted with an aminooxy-probe via oxime ligation.

A collection of labeling methodologies developed to selectively install a molecular probe on the N-terminal position of a protein target exploits indirect approaches that require two consecutive reaction steps (Figure 4). The first reaction step affords the selective modification of the N-terminal alpha-amine group into a not naturally occurring functionality (i.e., an aldehyde, a ketone, an azide, or an alkyne group). The second step is the actual conjugation reaction that provides the introduction of the desired probe through a chemo-selective reaction between the functionality installed on the protein N-terminus and a molecular probe bearing an orthogonal group. The aldehyde function is the reactive handle most frequently used to afford the indirect labeling of the protein N-terminus because such function is versatile and can be targeted with a wide range of orthogonal probes through several chemistries. A convenient strategy exploited to selectively oxidize the N-terminal alpha-amine group into an aldehyde or a ketone function is the pyridoxal 5′-phosphate (PLP)-mediated transamination reaction [42] (Figure 4a). The PLP is an aldehyde able to condensate with all the amine groups of a protein molecule (i.e., the N-terminal alpha-amine and the epsilon-amine of lysines), leading to the formation of imine derivatives. However, only the imine formed on the N-terminus can tautomerize due to the lower value of the pKa of the alpha-amine with respect to that of the lysine amine group. The resulting glyoxyl imine hydrolyzes, affording the formation of an aldehyde or a ketone group specifically at the N-terminus. These carbonyl handles can be further modified with aldehyde reactive probes such as alkoxyamine or hydrazide probes that react with aldehydes through oxime and hydrazone ligation, respectively. However, the PLP transamination reaction yield is not always high and shows a strong sequence dependence. Besides, the reaction is incompatible with some N-terminal amino acids. For instance, His, Trp, Lys, and Pro generate adducts with PLP while Cys and Ser undergo a beta-elimination side-reaction [82]. The reaction was used to prepare antibody conjugates functionalized at the N-terminal position with biotin, PEG, or Alexa dye alkoxyamine derivatives [83]. Protein labeling with PLP was carried out in an aqueous buffer at pH 6.5 at a temperature of 37–50 °C for 18–20 h in the presence of 10 mM PLP using the target antibody at low concentration. However, the yields of labeling were low and elevated temperatures were required to promote the reaction, limiting the general applicability of the approach. An alternative transamination reagent, the N-methylpyridinium-4-carboxaldehyde benzenesulfonate salt (Rapoport’s Salt) [84], resulted in being particularly effective for the labeling of glutamate-terminal proteins and thereby results in being useful for the site-selective modification of wild-type human IgG1 naturally possessing an N-terminal glutamic acid residue [43] (Figure 4a). N-terminal glutamate residues may also be exposed on recombinant proteins by proteolytic cleavage with Factor Xa [85]. A straightforward and rapid method for the installation of an N-terminal aldehyde function on proteins harboring a 1,2-aminoalchool function (i.e., a protein harboring an N-terminal Ser or Thr residue) refers to an oxidation reaction performed by treatment with sodium periodate (NaIO_4_) [72,86] (Figure 4b). The reaction is fast and requires mild conditions as it rapidly proceeds in an aqueous solution at neutral pH in the presence of a slight excess of NaIO_4_ and using very diluted reactant concentrations. However, a prolonged treatment with NaIO_4_ may lead to the undesired oxidation of other amino acids such as Met and Cys [86,87], hence the aldehyde protein should be quickly purified after the treatment with periodate in order to avoid side-reactions. The aldehyde function can be selectively targeted using aa chemo-selective reaction such as oxime ligation. We recently exploited such methodology to site-specifically modify the domain 2 of the vascular endothelial growth factor receptor 1 (VEGFR1D2) by introducing a functional probe at the protein N-terminus [44,88]. The serine at the N-terminus was selectively converted into an N-terminal glyoxalamide group by periodate mediated oxidation and subsequently reacted by oxime ligation with an oxyamine-biotin. N-terminal Ser oxidation with periodate was also combined to a strain-promoted alkyne–nitrone cycloaddition (SPANC) bioorthogonal reaction for the N-terminal site-specific modification of peptides and proteins, leading to N-alkylated isoxazolines as conjugation products [89]. In this three-step protocol, the N-terminal Ser protein substrate was subjected to oxidation with NaIO_4_ (1.1 equiv, 1 h) and, after treatment with p-methoxybenzenethiol, N-methylhydroxylamine and p-anisidine, was finally reacted with a cyclooctynol probe, yielding, after 24 h of incubation, the complete conversion of the protein target into the isoxazoline derivative via nitrone formation. The protocol was successfully used to prepare N-terminally pegylated Interleukin-8 using a PEG-cyclooctynol as the reagent. Indole probes able to react by the Pictet–Spengler reaction in mild conditions with the aldehyde protein have also been successfully used for N-terminal protein labeling [77,78].

**Figure 5:**
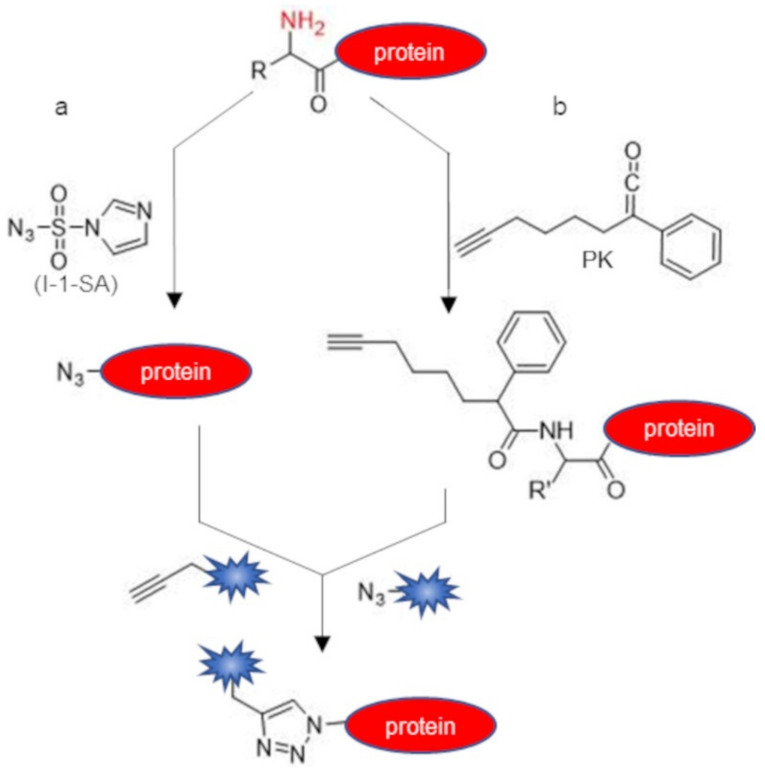
Two-step N-terminal protein labeling. (**a**) N-terminal alpha-amine group can be converted into an azide group by diazotransfer with imidazole-1-sulfonyl azide (I-1-SA); (**b**) acylation with phenyl ketene (PK) allows for the introduction of a N-terminal alkyne group. After the selective introduction of an alkyne or an azide function at the protein N-terminus, the protein can be further modified through copper-catalyzed [3 + 2] cycloaddition (CuAAC) click chemistry.

Besides aldehydes and ketones, the conversion of the N-terminal alpha-amine into an azide group is another straightforward way to introduce a targetable handle into proteins, as azides can be conveniently reacted using an alkyne-probe and click-chemistry reaction [90,91] (Figure 5a). N-terminal alpha-amine can be efficiently converted into azides through aqueous diazotransfer using the reagent imidazole-1-sulfonyl azide [45]. Performing the reaction at pH 8.5 allows for the selective azidation of the N-terminus over lysine residues. An alkyne function can also be installed at the protein N-terminus through the convenient use of a phenyl ketene reactive probe harboring an alkyne handle [46] (Figure 5b). The ketene probe developed was able to react with the alpha-amine group, leading to an amide conjugate and was demonstrated to show high selectivity against the N-terminal alpha amine of the majority of the natural amino acid residues. The strategy appeared compatible with the presence of other reactive groups such as the –OH group of Ser and Thr and –NH_2_ of Lys. However, the thiol group of Cys reacts with the ketene probe, leading to a thioester derivative that can be hydrolyzed by treatment with hydroxylamine. As proof of concept, the alkyne modified ketene probe was used to selectively label insulin, lysozyme, RNase A, and BCArg at the N-terminal position in aqueous solution. The copper-catalyzed [3 + 2] cycloaddition (CuAAC) reaction with a dansyl azide probe afforded protein labeling.

## 3. Enzymatic Labeling of Protein N-Terminus

**Figure 6:**
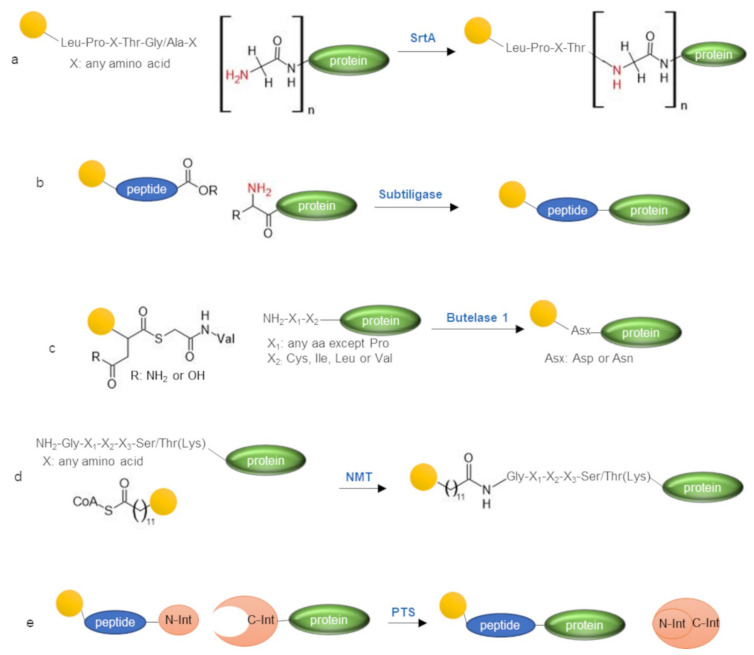
Enzyme-catalyzed N-terminal protein labeling. N-terminal protein modification mediated by (**a**) sortase A (SrtA); (**b**) subtiligase; (**c**) N- Myristoyltransferase (NMT); (**d**) butelase 1; (**e**) protein trans-splicing (PTS). Aa, amino acid; CoA, coenzyme-A; N-Int, N-terminal domain of a split intein; C-Int, C-terminal domain of a split intein.

Enzymatic approaches for N-terminal protein labeling represent a convenient alternative to the use of chemical reagents (Table 2, Figure 6). Enzymes ensure a uniquely high level of selectivity, perform in very mild reaction conditions, and need short reaction times. However, the use of enzymes in protein labeling may be expensive and consequently limited to a small scale. A number of enzymes, mainly transferases, have been exploited to selectively target protein N-terminus. They are used to catalyze the appending of a peptide, comprising the enzyme consensus sequence and site-specifically modified with the label of choice (such as fluorophores, biotin, lipids, nucleic acids, carbohydrates, and so on) at the N-terminus of the protein target.

### 3.1. Sortase A

Sortase A (SrtA) is one of the most widely explored enzymes for protein labeling [92] (Figure 6a). SrtA is a bacterial transpeptidase able to catalyze the ligation of a synthetic peptide reproducing the SrtA recognition sequence (Leu-Pro-X-Thr-Gly/Ala, where X is any amino acid, the fifth amino acid is Gly or Ala depending on the specific SrtA used) to an N-terminal oligo-Gly stretch introduced upstream of the protein target sequence [47,93,94]. SrtA exhibits a catalytic Cys able to cleave the peptide bond between the Thr and the Gly residues within the pentapeptide consensus sequence and form a thioacyl intermediate complex, which ultimately reacts with the N-terminal alpha-amine of an oligo-Gly protein, resulting in the formation of a new peptide bond between the protein and the peptide tag. SrtA can also be exploited to label protein C-terminus [92,95,96], internal loop regions [97,98], and for protein head-to-tail cyclization [99].

### 3.2. Subtiligase

Subtiligase is another powerful tool for the selective enzymatic modification of protein N-terminus [48,100] (Figure 6b). Subtiligase is a peptide ligase derived from the serine protease subtilisin through rational mutagenesis. By introducing two amino acid substitutions (Ser221Cys and Pro225Ala) into the subtilisin sequence, peptide ligation activity of the enzyme was effectively enhanced with respect to the parent protease activity, successfully yielding a powerful peptide ligation catalyst [101]. Subtiligase catalyzes the ligation of a C-terminal ester peptide to the N-terminal alpha-amine group of a protein target, with absolute selectivity over other amino acid nucleophiles. Using specifically modified C-terminal ester peptides, functional handles can be selectively introduced at the protein N-terminus. The C-terminal thioester peptide can also be exploited as subtiligase substrates, performing even better than ester peptides [102]. Reaction efficiency strongly relies on the N-terminal sequence and structure of the target protein. A set of subtiligase mutants with different sequence specificities is available and the most convenient subtiligase mutant can be selected according to the first two N-terminal amino acids of the protein substrate using the ALPINE (α-Amine Ligation Profiling Informing N-terminal modification Enzyme selection, https://wellslab.ucsf.edu/alpine) web application. Alternatively, a mixture of subtiligase mutants, showing a broad sequence specificity, can be conveniently employed [103]. Subtiligase can modify loop and β-sheet protein regions more efficiently than α-helices [104]. Therefore, if the N-terminus of the target protein is structurally organized into a helix, the protein target should be modified, extending the N-terminal sequence with residues endowed of low helical propensity [104]. The subtiligase mutant stabiligase can instead be adopted to label protein targets with a structurally inaccessible N-terminal region, being able to retain enzymatic activity in denaturant conditions in the presence of 0.1% sodium dodecyl sulfate or 4 M guanidinium chloride [104].

### 3.3. Butelase 1

Butelase 1 is the fastest known Asn/Asp-specific peptide ligase isolated from the medicinal plant Clitoria ternatea and mediates, in vivo, the backbone cyclization in the biosynthesis of cyclotides [105]. Butelase 1 recognizes the tripeptide Asn/Asp-His-Val at the C-terminus of the target and mediates backbone cyclization by cleaving the consensus sequence between Asn/Asp and His and ligating the Asn/Asp residue to the N-terminal alpha-amine to form a macrocycle. Although mainly proposed for protein macrocyclization [106], butelase 1 was also efficiently adopted for peptide ligation and N-terminal protein labeling [49]. Conveniently, butelase 1 accepts any amino acid at the N-terminal protein target position, except Pro, to form a new Asn/Asp-X peptide bond. However, it exhibits a more stringent amino acid requirement at the second position, showing a strong preference for Cys and the hydrophobic amino acids Ile, Leu, and Val [105]. Butelase 1-mediated intramolecular cyclization proceeds with high efficiency and is an irreversible reaction. In contrast, the intermolecular peptide ligation reaction catalyzed by butelase 1 is a reversible reaction that requires an excess of substrate to reach completion [105]. Using Asn/Asp-thiodepsipeptide as a substrate, which releases a thiol that is a poor competing nucleophile and is not recognizable by butelase 1, the ligation reaction becomes irreversible, allowing the effective use of butelase 1 in peptide ligation and the N-terminal protein labeling reaction (Figure 6c) [49]. A similar behavior was also observed for SrtA-mediated labeling, in which case, the use of depsipeptide substrates enabled the ligation reaction to efficiently procced using equimolar quantities of substrates and substoichiometric quantities of SrtA [107].

### 3.4. N-Myristoyltransferase

N-Myristoyltransferase (NMT) is an eukaryotic enzyme devoted to catalyzing the acylation with myristic acid (using myristoyl-CoA as the activated reagent) of the alpha-amine group of proteins exposed at the N-terminus of the NMT recognition sequence Gly-X1-X2-X3-Ser/Thr (Lys) (X is any amino acid) (Figure 6d). NMT can also use surrogates of the myristic acid as a substrate [108,109,110] such as azido or alkyne containing fatty acids, allowing the N-terminal site-specific introduction of an orthogonal reactive handle that can be conveniently exploited for protein decoration using Staudinger ligation [111], CuAAC, or strain-promoted azide-alkyne cycloaddition (SPAAC) click-chemistries [50,112,113]. A protocol for the recombinant co-expression in *Escherichia coli* of NMT and the protein target for the in vivo N-terminal labeling with a clickable myristic acid analog was also provided [114].

### 3.5. Protein Trans-Splicing

Protein trans-splicing (PTS) can also be employed to afford N-terminal protein labeling [24,115,116] (Figure 6e). PTS takes advantage of a particular class of inteins, called split inteins, that, if divided into two domains, can reassemble non-covalently in solution into a functional intein able to catalyze protein splicing by mediating its self-cleavage and ligation of the two exteins. Some inteins such as Ssp DnaB can be split at an amino acid position along the intein sequence that is very close to the N-terminus [117], rendering the N-terminal fragment of the split intein so short that it can be prepared by the chemical route using solid-phase peptide synthesis (SPPS), thus allowing the incorporation of any desired modification such as the introduction of a functional label on the N-terminal alpha-amine group.

## 4. Conclusions

In summary, a wide collection of chemical reactions are available for the site-specific modification of a protein through the targeting of the N-terminal alpha-amine group. The plethora of methodologies available, that have been described herein with their advantages and disadvantages, allow for the decoration of protein targets with very different properties. The labeling strategies can be grouped in direct and indirect approaches, respectively requiring one or two reaction steps to afford N-terminal protein decoration. Indirect strategies appear to be more versatile methodologies with respect to the kind of probe to be introduced as they allow for the functionalization of the protein target with a reactive handle that can be selectively targeted with a wide collection of commercially available molecular probes. In contrast, direct approaches, although being more rapid as the protein modification is accomplished in a single reaction step, require a greater synthetic effort for the preparation of the molecular probe that should be specifically tailored. The more appropriate chemical strategy to be adopted among all those available should be chosen according to the properties of the protein target (especially the type of native N-terminal amino acid) and of the handle to be introduced. Of note, methods based on the control of the reaction pH should be carefully evaluated as, in a protein, the pKa of a functional group could be, sometimes, very different from the expected one because of the influence of the chemical environment.

## Figures and Tables

**Table 1:** Chemical strategies developed for site-specific protein bioconjugation through the targeting of the N-terminal position.

Chemical Reagent	Target Function	Reaction	Junction	Experimental Conditions ^a^	Yield of Conversion (%) ^a^	Ref
benzaldehydes	α-amine	reductive amination	secondary amine	citric acid buffer pH 6.1, 2 eq of benzaldehyde, 5 eq ^b^ of NaBH_3_CN, 6–48 h, r.t.	30–70	[29]
2-ethynylbenzaldehydes (2-EBA)	α-amine	imine formation and intramolecular 6-endo-dig cyclization	isoquinolinium	PBS/DMSO 9:1 pH 6.5–7.4, 5–10 eq of 2-EBA, 1–16 h, 37 °C	10–92	[30]
N-hydroxy-phthalimide (NHP)	α-amine	phthalimidation	phthalimidoamine	phosphate buffer pH 7.0, 1 eq of NHP, 8 h, r.t.	45–50	[31]
selenobenzaldehyde (SBA) ester	α-amine	acylation through ACL	amide	dimethylformamide/PBS pH 7.0, 5 eq of SBA ester, 40 h, r.t.	70	[32]
thioesters	1,2-aminothiol (N-term Cys)	acylation through NCL	amide	phosphate buffer pH 7.4, 20 eq mercaptoethanesulfonate 4 eq of thioester probe, 5 h, r.t.	~100	[33]
2-cyanobenzothiazole (CBT)	1,2-aminothiol (N-term Cys)	condensation	thiazoline	PBS pH 7.4, 2 mM glutathione or TCEP, 5 eq of CBT, 2 h, r.t.	Not reported	[34]
aldehydes (e.g., glyoxalaldhehyde)	1,2-aminothiol (N-term Cys) or 1,2-aminoalcohol (N-term Ser/Thr)	condensation	thiazolidine or oxazolidine	acetate buffer pH 4.5, 1 mM DTT or TCEP 16 h, then 40–200 eq aldehyde, 2.5−4 days, 4 °C	90	[35]
2-formyl phenylboronic acid (2-FPBA)	1,2-aminothiol (N-term Cys)	condensation	thiazolidino-boronate	phosphate buffer pH 7.0, 1 eq of 2-FPBA 30 min, r.t.	~100	[36]
aldehydes	N-term Trp	Pictet–Spengler condensation	tetrahydro-beta-carboline	Glacial acetic acid, 24 h reagents ratio not reported	~100	[37]
2-pyridinecarbaldehyde (2-PCA)	α-amine	imine condensation	imidazolidinone	phosphate buffer pH 7.5, 25 μM protein substrate, 10 mM 2-PCA, 37 °C, 16 h	43–95	[38]
ortho-aminophenols	α-amine (especially N-term Pro)	oxidative coupling	secondary or tertiary amine ^c^	phosphate buffer pH 7.5, 20 μM protein substrate, 5 eq of ortho-aminophenol, 5 mM K_3_Fe(CN)_6_, 30 min, r.t.	up to ~ 100 (on N-term prolyl-protein)	[39]
2-(2-formylphenoxy)acetic acid (2-FPOAA)	α-amine of N-term Gly	imine condensation + nucleophilic addition	amino-alcohol	bicarbonate buffer pH 7.8, 20 μM protein substrate 10 mM 2-FPOAA, 24–48 h, 20 °C	40–71	[40]
4-methoxyphenyl esters (4-MOPE)	α-amine of Gly-His_n_ N-term tag	acylation	amide	HEPES buffer pH 7.5, 20 eq of 4-MOPE, 24 h, 4 °C	45 (on Gly-His_6_ tagged protein)	[41]
pyridoxal-5′-phosphate (PLP)	α-amine	transamination	aldehyde/ketone	phosphate buffer pH 6.5, 10 mM PLP, 18–20 h, 37–55 °C	30–80	[42]
Rapoport’s salt (RS)	α-amine (especially N-term Glu)	transamination	aldehyde/ketone	phosphate buffer pH 6.5, 100 mM RS 1 h, 37 °C	67	[43]
sodium periodate (NaIO_4_)	1,2 amino-alcohol (N-term Ser or Thr)	oxidation	aldehyde	phosphate buffer/NaCl pH 7.0, 1.5 eq, of NaIO_4_, 1 h, r.t.	~100	[44]
imidazole-1-sulfonyl azide (I-1-SA)	α-amine	diazotransfer	azide	diethanolamine buffered solution pH 8.5, 17.5 eq of I-1-SA, overnight, r.t.	~100	[45]
phenyl ketene (PK)	α-amine	acylation	amide	phosphate buffer pH 6.3 or 9.2, 6–10 eq of PK, 15 min—overnight, 37 °C	23–38	[46]

^a^ Yields of conversion and reaction conditions are reported according to the cited references. Of note, these parameters strongly rely on the protein targets and on the type of probe used as labeling reaction substrates and therefore are not universal values but should vary according to protein substrate and probe properties. ^b^ Reagent equivalents are calculated with respect to the protein substrate. ^c^ A tertiary amine is obtained if the reaction is performed on the N-terminal prolyl-protein. PBS, phosphate buffer saline; r.t., room temperature; GuHCl, guanidinium chloride; TCEP, tris-carboxyethylphosphine; DTT, dithiothreitol; ACL, aldehyde capture ligation; NCL, native chemical ligation; K_3_Fe(CN)_6,_ potassium ferricyanide.

**Table 2:** Enzyme-catalyzed strategies for site-specific N-terminal protein labeling.

Enzyme	Reagent	N-terminal Target Sequence or Function	Reaction Conditions ^a^	Yield ^a^	Ref
Sortase A (SrtA)	Leu-Pro-X-Thr-Gly/Ala-X peptide (X any a.a.)	α-amine of oligo-Gly	0.5–1 mM probe-Leu-Pro-X-Thr-Gly/Ala-, 10–50 µM target protein, 20–150 µM SrtA, in 50 mM Tris buffer pH 7.5, 150 mM NaCl, 10 mM CaCl_2_ 15 min—5 h, r.t. or 37 °C	up to 90	[47]
Subtiligase	C-terminal ester peptide	α-amine	5 mM peptide ester-probe, 10–50 µM target protein, 1 µM subtiligase, in 100 mM tricine pH 8.0, 1–2 h, 4 °C or r.t.	variable ^b^	[48]
Butelase 1	Thiodepsipeptide Asn/Asp-(S) Gly-Val	α-amine of X_1_-X_2_ (X_1_ any a.a. except Pro; X_2_ Cys, Ile, Leu, Val)	100 µM target protein, 0.1 µM butelase 1, 400–500 µM thiodepsipeptide (1 eq added every 30 min) 1 mM EDTA, 20 mM phosphate buffer pH 6.5, 100 min—2.5 h, temperature not specified	up to 95	[49]
N-Myristoyltransferase (NMT)	myristoyl-CoA derivatives	α-amine of Gly-X_1_-X_2_-X_3_-Ser/Thr (Lys) (X is any amino acid)	15 µM target protein, 2 mM DTT 30 µM myristoyl-CoA derivative in 30 mM Tris buffer, 0.5 mM EGTA, 0.1% TRITON^®^ X-100, pH 7.4 18 h—37 °C	not reported	[50]

^a^ Yields of conversion and reaction conditions are reported according to the cited references. These parameters are not universal values but could vary according to the substrates’ properties. ^b^ Dependent on the N-terminal target protein sequence and structure. EDTA, ethylenediaminetetraacetic acid; a.a., amino acid; r.t., room temperature; DTT, dithiothreitol; EGTA, ethylene glycol-bis(2-aminoethylether)-N,N,N′,N′-tetraacetic acid.

## Data Availability

Not applicable.
